# Time to Complete Clinical Recovery and Its Predictors in Bell’s Palsy Patients Receiving Acupuncture: A Prospective Cohort Study

**DOI:** 10.3390/medicina62071248

**Published:** 2026-06-29

**Authors:** Aleksandar Kopitović, Sandro Kalember, Filip Katanić, Nina Vico Katanić, Zita Jovin, Sofija Banić Horvat, Miroslav Ilin, Marko Bojović, Svetlana Simić

**Affiliations:** 1 Faculty of Medicine, University of Novi Sad, 3 Hajduk Veljkova Street, 21000 Novi Sad, Serbia; aleksandar.kopitovic@mf.uns.ac.rs (A.K.); katanic.f@gmail.com (F.K.); nina.vico@gmail.com (N.V.K.); marko.bojovic@mf.uns.ac.rs (M.B.); svetlana.simic@mf.uns.ac.rs (S.S.); 2 Department of Neurology, University Clinical Center of Vojvodina, 1-9 Hajduk Veljkova Street, 21000 Novi Sad, Serbia; zitajovin@yahoo.com (Z.J.); sofijabanichorvat@gmail.com (S.B.H.); mmtsilin@gmail.com (M.I.); 3 Special Gynecology Hospital Ferona, 19 Sarplaninska Street, 21000 Novi Sad, Serbia; 4 Department of Anesthesia, Intensive Care and Pain Therapy, University Clinical Center of Vojvodina, 1-9 Hajduk Veljkova Street, 21000 Novi Sad, Serbia; 5 Oncology Institute of Vojvodina, Put Doktora Goldmana 4, 21204 Sremska Kamenica, Serbia

**Keywords:** Bell’s palsy, acupuncture, electromyography, compound muscle action potential, prognosis, time-to-event analysis, facial nerve regeneration

## Abstract

*Background and Objectives*: Bell’s palsy (BP) is the most common cause of acute unilateral peripheral facial nerve paralysis. The aim of this study was to evaluate the time to complete clinical recovery in patients with BP treated with acupuncture and to identify baseline clinical and electrophysiological predictors of recovery outcomes. In addition, electrophysiological characteristics at the time of complete clinical recovery were examined. *Materials and Methods*: This prospective, observational, uncontrolled cohort study included 1050 patients with clinically confirmed BP who received acupuncture as the only therapeutic intervention between January 2017 and August 2025. Clinical severity was assessed using the House–Brackmann (HB) and Sunnybrook (SB) grading systems. Electrophysiological evaluation included compound muscle action potential (CMAP) analysis and needle electromyography (EMG). Time-to-event analysis was performed using the Kaplan–Meier method and Cox proportional hazards regression analysis. *Results*: Complete clinical recovery was achieved in 843 patients (80.3%). The median time to recovery was 40 days (IQR 30–60). Patients with milder baseline deficits (HB II–IV) demonstrated significantly faster recovery than those with severe paralysis (HB V–VI) (log-rank *p* < 0.001). In multivariable Cox regression analysis, higher baseline HB grade, older age, and more severe EMG denervation were independently associated with slower recovery. Residual electrophysiological abnormalities persisted in most patients despite complete clinical recovery. *Conclusions*: Recovery from BP is a dynamic and heterogeneous process, significantly influenced by initial clinical severity and the degree of electrophysiological impairment. Combined clinical and electrophysiological assessment may contribute to more precise prognostic stratification and follow-up of patients with BP. Persistent electrophysiological abnormalities despite complete clinical recovery suggest that complete clinical recovery may precede complete neurophysiological regeneration of the facial nerve.

## 1. Introduction

Bell’s palsy (BP) is the most common cause of acute unilateral peripheral facial nerve paralysis, with an estimated annual incidence of 15–30 cases per 100,000 population [1,2]. The exact cause of BP is still unclear. However, the leading hypothesis is that reactivation of latent herpes simplex virus within the geniculate ganglion leads to inflammation, nerve edema, microvascular compromise, and subsequent demyelination or axonal injury [3]. The condition is typically characterized by a sudden onset of facial weakness that reaches maximal severity within 48–72 h. Although spontaneous recovery occurs in the majority of patients, approximately 15–30% experience residual deficits, including incomplete motor recovery, facial asymmetry, synkinesis, contractures or chronic neuropathic symptoms [4]. Current evidence suggests that the underlying pathophysiology involves inflammation-induced edema of the facial nerve within the confined space of the Fallopian canal, leading to ischemia, demyelination, and, in more severe cases, secondary axonal degeneration [5,6].

Systemic corticosteroids initiated during the early stage of the disease are considered the cornerstone of treatment for BP. Multiple randomized clinical trials have shown that oral prednisolone administered within the first 72 h after symptom onset significantly increases the likelihood of achieving complete recovery [7,8]. This time-dependent therapeutic window is critical, as corticosteroids exert their maximal effect during the early inflammatory phase, characterized by neural edema and compression within the facial canal. Consequently, contemporary evidence-based clinical guidelines strongly recommend initiating corticosteroid therapy within this 72 h interval. After this period, the magnitude of therapeutic benefit declines substantially, particularly following the development of secondary axonal degeneration [9,10,11].

Acupuncture has been commonly used in the management of BP, either as a complementary intervention alongside conventional therapy or as an independent treatment modality, particularly in East Asian countries. Several systematic reviews and meta-analyses have investigated the potential role of acupuncture in BP management and have reported associations with improved facial motor outcomes and facial symmetry compared with standard treatment approaches. However, the overall quality of evidence remains limited by methodological heterogeneity, risk of bias, and variability in study design [12,13]. The proposed biological mechanisms include improved regional microcirculation, regulation of inflammatory signaling pathways, and promotion of neuroplastic remodeling within the facial nerve nucleus and its peripheral branches [14].

Although corticosteroids remain the standard treatment for acute Bell’s palsy, a proportion of patients either present outside the recommended therapeutic window (72 h), do not receive corticosteroid therapy before specialist evaluation, or seek complementary treatment approaches as part of routine clinical practice. Consequently, cohorts of patients managed without prior corticosteroid exposure continue to be encountered in specialized acupuncture settings. Studying such populations may provide valuable insights into recovery patterns and the interplay between clinical and electrophysiological predictors of recovery in patients receiving acupuncture, while not implying comparative treatment efficacy [12,13,14]. Despite these findings, significant methodological limitations persist in the current literature. Most studies continue to rely primarily on clinical grading systems, with limited use of objective neurophysiological assessments. While electroneurography and compound muscle action potential (CMAP) analysis are established for early prognostic evaluation of facial nerve impairment, serial needle electromyography (EMG) is particularly valuable for monitoring ongoing denervation, reinnervation, and motor unit changes. However, longitudinal EMG monitoring is seldom incorporated into acupuncture studies, which restricts understanding of the neurobiological mechanisms underlying functional recovery. As a result, the relationship between clinical improvement and electrophysiological regeneration remains unclear [15].

In this study, the term “recovery dynamics” refers to the temporal pattern of complete recovery, including time to complete clinical recovery and its relationship with baseline clinical and electrophysiological characteristics.

The aims of the study:

1. Evaluation of time to complete clinical recovery in patients with BP treated with acupuncture and identification of baseline clinical and electrophysiological factors associated with recovery outcomes.
2. Exploratory assessment of electrophysiological findings at the time of complete clinical recovery.

## 2. Materials and Methods

This prospective, observational, uncontrolled cohort study was conducted at the Clinic of Neurology, University Clinical Center of Vojvodina, from January 2017 to August 2025. No a priori sample size calculation was performed. All consecutive eligible patients presenting during the study period were considered for inclusion, and the final sample size was determined by the number of patients meeting the predefined eligibility criteria. Patients were treated at the Acupuncture Outpatient Clinic, established in 1979, which has longstanding experience in managing peripheral facial palsy. Patients were referred to the Acupuncture Outpatient Clinic either by other physicians or through self-referral as part of routine clinical practice. Due to the clinic’s longstanding clinical experience in managing peripheral facial palsy, referral patterns have developed over time, resulting in a substantial number of patients being evaluated and treated in this setting. The study was performed in accordance with the principles of the Declaration of Helsinki. The study protocol was approved by the Ethics Committee of the Clinical Center of Vojvodina (Protocol No. 00–81/1121; approved on 30 December 2016). All participants provided written informed consent for participation and prospective collection of clinical and electrophysiological data in accordance with institutional regulations.

The study included 1050 patients with clinically confirmed BP who received acupuncture as the only therapeutic intervention. Because the study was observational in nature and did not involve assignment to specific treatment groups, only patients without prior treatment for Bell’s palsy were eligible for inclusion.

### 2.1. Inclusion Criteria

- Diagnosis of unilateral idiopathic BP (International Classification of Diseases (ICD), 10th and 11th Revision);
- Acute phase of the disease, defined as symptom onset within 7 days before the initial evaluation;
- First episode of peripheral facial nerve paralysis;
- Treatment with acupuncture as the only treatment modality used;
- Availability of a complete baseline clinical and electrophysiological assessment performed before treatment initiation.

### 2.2. Exclusion Criteria

- Facial paralysis of non-idiopathic etiology, including Guillain–Barré syndrome, Lyme disease, Ramsay Hunt syndrome, Sarcoidosis, cholesteatoma, parotid gland tumors or other neoplasms involving the facial nerve, traumatic facial nerve injury, iatrogenic facial nerve injury;
- Diabetes mellitus, because it is a recognized prognostic factor that may independently influence peripheral nerve regeneration and functional recovery after Bell’s palsy, thereby potentially confounding the evaluation of recovery predictors;
- Central nervous system pathology suggestive of central facial weakness or other structural CNS causes;
- Bilateral facial nerve involvement;
- Recurrent episodes of facial nerve paralysis;
- Previous surgical intervention involving the facial nerve or other invasive procedures in the facial nerve region;
- Systemic neurological disorders affecting cranial nerve function;
- Previous treatment for facial nerve paralysis before presentation at the institution, including systemic corticosteroids, antiviral therapy, or any other specific pharmacological or non-pharmacological treatments;
- More than 7 days elapsed before the initial evaluation;
- Incomplete baseline clinical or electrophysiological data.

### 2.3. Clinical Assessment

The diagnosis of BP was established by two neurologists with extensive clinical experience in peripheral facial nerve disorders, based on clinical examination and the exclusion of secondary causes of facial nerve dysfunction.

Disease severity was assessed using the House–Brackmann (HB) and Sunnybrook (SB) Facial Grading Systems. Baseline evaluation was performed before treatment initiation and repeated after completion of each treatment cycle.

The HB scale classifies facial nerve dysfunction into six ordinal grades (I–VI), ranging from normal facial function to complete paralysis [4]. The SB scale provides a regionally weighted composite score (0–100) that integrates resting symmetry, voluntary facial movement, and synkinesis [16]. Both instruments are validated and widely accepted for assessing peripheral facial nerve dysfunction.

Clinical assessments using the House–Brackmann and Sunnybrook grading systems were performed by neurologists experienced in evaluating and managing facial nerve disorders. All assessors were familiar with the application of both grading systems through routine clinical practice. Formal inter-rater reliability testing was not performed.

Complete clinical recovery was defined as House–Brackmann grade I. For the purposes of prognostic and survival analyses, baseline HB grade was dichotomized into mild-to-moderate (HB ≤ 4) and severe (HB ≥ 5) disease according to initial clinical severity.

### 2.4. Electrophysiological Assessment

Electrophysiological evaluation was performed using a standardized protocol that included bilateral assessment of CMAP and needle EMG.

CMAP recordings were obtained from the orbicularis oculi muscle using surface electrodes following transcutaneous stimulation of the facial nerve in the preauricular/stylomastoid region. Measurements were recorded as peak-to-peak amplitudes on both the affected and contralateral sides. To reduce interindividual variability, CMAP amplitudes were expressed as the ratio of the affected to the contralateral side ((affected/contralateral) × 100), a commonly used method in electrophysiological assessment of the facial nerve [17].

Needle EMG examinations were performed bilaterally on the orbicularis oculi and orbicularis oris muscles. EMG findings at each time point were quantified using separate variables. The degree of denervation was assessed using an ordinal scale of spontaneous activity intensity (0–4) based on standard electrodiagnostic criteria [18,19].

Baseline electrophysiological assessment was conducted before treatment initiation. To minimize interobserver variability, all electrophysiological measurements were performed according to a predefined standardized protocol. Interpretation of the findings was performed by three experienced neurophysiologists with extensive clinical experience in the electrodiagnostic evaluation of peripheral neuropathies. Assessment of denervation was based on clearly defined electrodiagnostic criteria. In cases of equivocal or borderline findings, the final interpretation was established by consensus among the investigators. An additional electrophysiological assessment was performed at the time of complete functional recovery.

Electrophysiological examinations were performed using a five-channel Synergy EDX EMG system (Natus Medical Inc., Middleton, WI, USA).

### 2.5. Acupuncture Intervention Protocol

All patients received a standardized acupuncture treatment protocol. Sterile disposable stainless-steel filiform needles (0.25 mm diameter; lengths 13–40 mm) were used. Manual acupuncture was used exclusively; electroacupuncture was not used. Gentle manual stimulation was performed after needle insertion to elicit deqi sensation, characterized by heaviness, fullness, numbness, tingling, or a mild radiating sensation without significant pain. Treatments were administered according to a standardized protocol by physicians trained in medical acupuncture and experienced in managing neurological disorders.

Needle insertion depth and angle were adapted to the anatomical characteristics of each acupuncture point, with insertion depths ranging from 5 to 30 mm. The following points were consistently targeted: Renzhong (GV26), Heliao (LI19), Dicang (ST4), Jiache (ST6), Sibai (ST2), Tinggong (SI19), Zanzhu (BL2), Yintang (EX-HN3), Fengchi (GB20), Hegu (LI4), Neiting (ST44), and Taichong (LR3).

A completed STRICTA 2010 checklist and a detailed description of the acupuncture intervention are provided in the Supplementary Materials (Supplementary Tables S1 and S2) [20].

### 2.6. Treatment Protocol and Follow-Up

**Figure 1:**
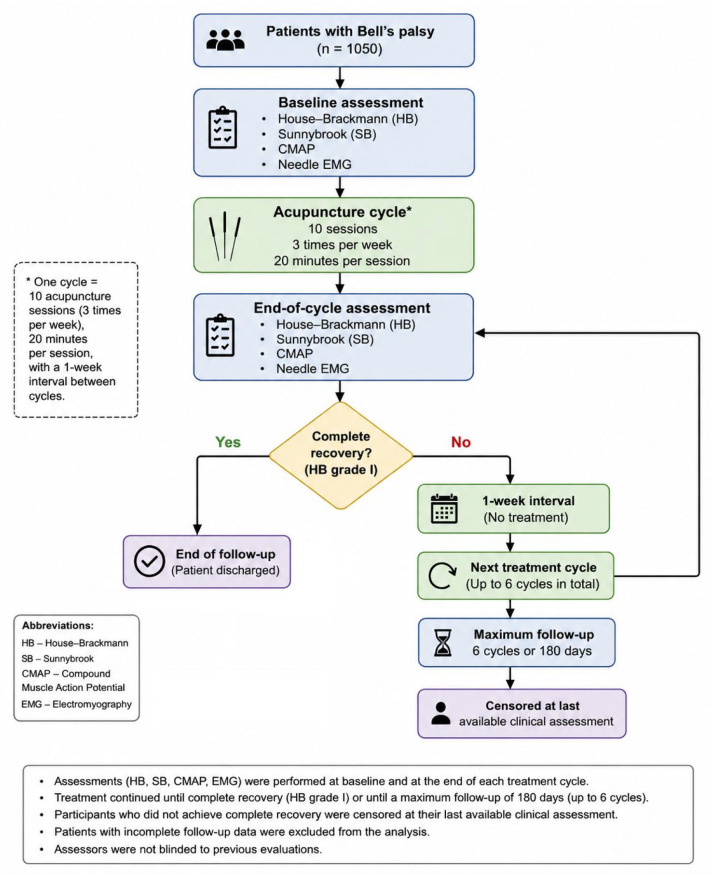
Study design and follow-up workflow.

Acupuncture treatment was administered in cycles consisting of 10 sessions performed three times weekly, with each session lasting approximately 20 min. A one-week interval was scheduled between consecutive treatment cycles. Treatment was continued until complete recovery or a maximum follow-up period of 180 days (up to six treatment cycles). The overall study workflow and follow-up schedule are illustrated in Figure 1.

Clinical assessment using the House–Brackmann (HB) and Sunnybrook (SB) grading systems, together with electrophysiological evaluation including compound muscle action potential (CMAP) measurements and needle electromyography (EMG), was performed at baseline and repeated after each treatment cycle. For patients who achieved complete clinical recovery, the electrophysiological findings obtained at the assessment documenting recovery were used for the final analysis.

Participants who did not achieve complete recovery during the 180-day follow-up period were censored at their last available clinical assessment. Patients with incomplete follow-up data were excluded from the analysis. Due to the observational design and longitudinal follow-up, assessors were not blinded to previous clinical and electrophysiological evaluations.

### 2.7. Statistical Analysis

Statistical analyses were conducted using IBM SPSS Statistics, version 26.0 (IBM Corp., Armonk, NY, USA) and jamovi version 2.3.28 (The jamovi project, Sydney, Australia).

Continuous variables were presented as mean ± standard deviation or as median with interquartile range (IQR), depending on the data distribution. Categorical variables were presented as frequencies and percentages. Normality of continuous variables was assessed using the Shapiro–Wilk test. Because most continuous variables were not normally distributed, comparisons between groups were performed using the Mann–Whitney U test. Categorical variables were compared using Pearson’s χ^2^ test. Baseline clinical and electrophysiological characteristics were compared between patients who achieved complete recovery and those who did not.

Time-to-event analysis was performed using the Kaplan–Meier method, while differences between groups were assessed using the log-rank test.

The primary endpoint was the time to complete functional recovery, defined as the interval in days from treatment initiation to the first clinical assessment documenting complete resolution. Participants who did not achieve complete recovery during follow-up were censored at their last available clinical assessment.

Cox proportional hazards regression analysis was used to evaluate factors associated with time to recovery. Univariate Cox regression models were initially used to assess associations between individual baseline variables and recovery outcome, including age, sex, time from symptom onset to treatment initiation, baseline HB grade, baseline CMAP values, and degree of EMG denervation. Because the HB and SB scales assess overlapping aspects of facial nerve dysfunction, only HB grade was included in the multivariable model to avoid potential collinearity between clinical severity measures. Variables with *p*-values < 0.10 in the univariate analysis, as well as clinically relevant variables, were included in the multivariable model to identify factors independently associated with recovery. The proportional hazards assumption was evaluated using Schoenfeld residuals and graphical inspection of residual plots. Statistical significance was set at *p* < 0.05.

Results were reported as hazard ratios (HRs) with 95% confidence intervals (CIs). HR values greater than 1 indicated a higher likelihood of earlier recovery, while HR values less than 1 indicated slower recovery. Statistical significance was defined as *p* < 0.05.

## 3. Results

A total of 1050 patients were included in the analysis, of whom 843 (80.3%) achieved complete clinical recovery, while 207 (19.7%) did not. Patients who achieved complete recovery were significantly younger than those who did not (41 [IQR 30–52] vs. 56 [IQR 48–63] years, *p* < 0.001). No significant difference in sex distribution was observed between the groups (57.3% vs. 50.7% female patients, *p* = 0.103). Likewise, the time from symptom onset to treatment initiation did not differ significantly between the groups (median 4 [IQR 2–5] days vs. 3 [IQR 2–5] days, *p* = 0.76).

At baseline assessment, patients who achieved complete recovery exhibited significantly milder facial nerve dysfunction, as evidenced by a distinctly different distribution of HB grades between groups (χ^2^ = 392.9, *p* < 0.001). Severe disease (HB V–VI) was markedly more prevalent in the non-recovered group (88.4% vs. 17.0%), whereas milder forms (HB II–IV) were predominant among those who recovered (83.0% vs. 11.6%). Baseline SB scores were significantly higher in the recovery group (median 39.0 [IQR 26.0–57.0] vs. 20.0 [IQR 14.0–32.0]; *p* < 0.001), reflecting superior initial facial nerve function. Likewise, baseline CMAP values were significantly greater among patients who recovered (34.0% [IQR 23.0–50.0] vs. 15.0% [IQR 8.0–22.5]; *p* < 0.001), indicating better preservation of facial nerve electrophysiological integrity. The distribution of EMG denervation grades differed significantly between the two groups (χ^2^ = 408.5, *p* < 0.001), with grade 4 denervation most prevalent in the non-recovered cohort. A detailed overview of baseline patient characteristics is presented in Table 1.

For the 207 patients who did not achieve complete clinical recovery during follow-up, functional status at the last available assessment was further evaluated. The majority were classified as HB III (*n* = 146; 70.5%), with 25 (12.1%) classified as HB II, 32 (15.5%) as HB IV, and 4 (1.9%) as HB V.

**Figure 2:**
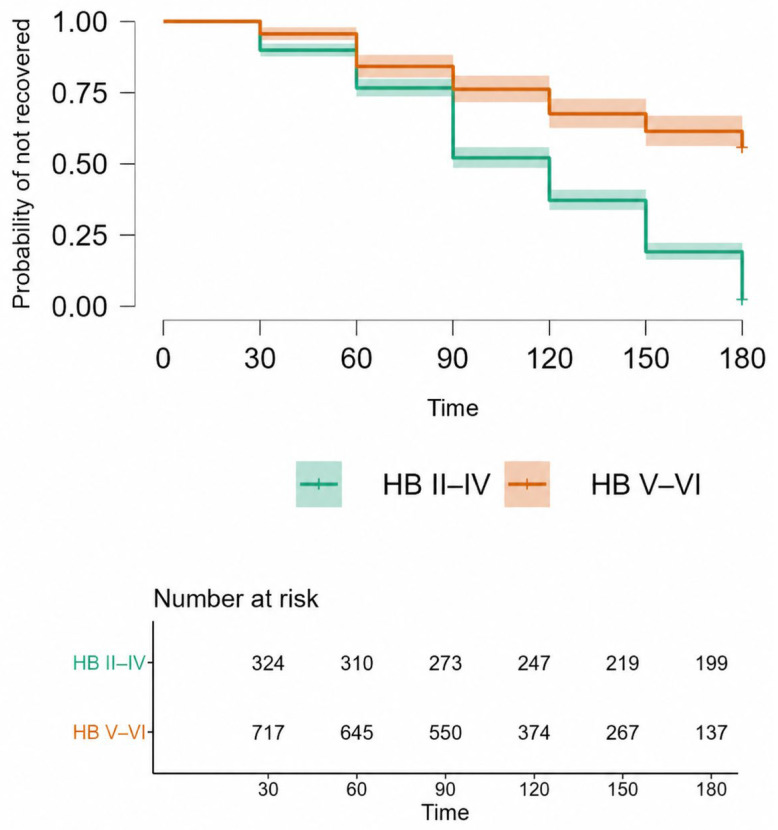
Kaplan–Meier curves showing time to complete recovery stratified by baseline House–Brackmann grade. Patients with milder palsy (HB II–IV) demonstrated significantly faster recovery compared to those with severe palsy (HB V–VI) (log-rank *p* < 0).

Kaplan–Meier analysis of time to complete recovery is shown in Figure 2. The median time to complete recovery was 40 days (IQR 30–60). Recovery dynamics differed significantly by baseline HB severity groups (HB II–IV vs. HB V–VI; log-rank *p* < 0.001). Patients with milder baseline dysfunction recovered more quickly and more often, whereas those with severe initial facial paralysis had a markedly reduced likelihood of achieving complete recovery throughout follow-up.

To identify factors associated with time to complete recovery, Cox proportional hazards regression analysis was performed. In a univariate Cox regression analysis, older age, higher baseline HB grade, lower CMAP values, male sex, and more severe electromyographic denervation were significantly associated with slower recovery. In contrast, the time from symptom onset to treatment initiation was not.

In the multivariate Cox regression model, higher baseline HB grade, older age, and more severe EMG denervation remained independently associated with slower recovery. Baseline CMAP values, analyzed as a continuous variable, were significantly associated with faster recovery in univariate analysis. However, after adjustment for clinical and electrophysiological parameters, this association was no longer statistically significant, suggesting that CMAP was not an independent predictor of recovery (Table 2). Assessment of the proportional hazards assumption using Schoenfeld residuals showed no significant violations for age or EMG denervation grade. A significant deviation was observed for baseline HB category, suggesting a modest time-varying effect. However, visual inspection of Schoenfeld residual plots and Kaplan–Meier curves did not reveal substantial departures from proportionality, and the Cox model was therefore retained with cautious interpretation of the HB effect.

### Electrophysiological Findings at the Time of Recovery

Electrophysiological data at the point of complete clinical recovery were available for 843 patients. At recovery, the median CMAP ratio was 48.0% (IQR 37.8–57.1). Residual EMG evidence of denervation persisted in 841 patients (99.8%), whereas reinnervation was observed in 699 patients (82.9%).

As shown in Table 3, patients with severe baseline facial palsy (HB V–VI) exhibited significantly lower CMAP ratios at clinical recovery compared to those with milder palsy (43.5% vs. 49.5%, *p* < 0.001). Residual EMG denervation was also more pronounced in the severe group (median score 1.42 vs. 1.00, *p* < 0.001). Conversely, reinnervation signs were more frequently observed in patients with initially severe palsy (89.3% vs. 81.1%, *p* = 0.014).

**Table 3:** Electrophysiological findings at clinical recovery according to baseline HB severity.

Variable	HB II–IV	HB V–VI	*p*-Value
Final CMAP %	49.5 [37.8–57.1]	43.5 [32.4–53.6]	<0.001 ^a^
Final EMG	1.00 [1.00–2.00]	1.42 [1.00–2.00]	<0.001 ^b^
Reinnervation present, n (%)	553 (81.1%)	146 (89.3%)	0.014

Legend: CMAP—Compound Muscle Action Potential; EMG—Electromyography Data are presented as median (IQR) or *n* (%). Statistic test: ^a^—Mann–Whitney U test; ^b^—Chi-square test.

## 4. Discussion

In this large prospective cohort study, more than 80% of patients with BP who underwent acupuncture treatment achieved complete clinical recovery, with a median recovery time of 40 days. Survival analysis showed that most recoveries occurred during the initial phase after treatment initiation, whereas the probability of recovery gradually decreased over time. These findings indicate marked temporal variability in the recovery process and further emphasize the importance of the early stage of the disease for functional regeneration of the facial nerve.

The high proportion of patients who achieved complete clinical recovery in our cohort is in line with previous studies reporting a generally favorable prognosis for BP [21]. Nevertheless, examining recovery duration provides a more comprehensive understanding of disease behavior than studies that assess only final clinical outcomes. Our results indicate that recovery does not follow a uniform or linear pattern but varies substantially among patients, likely due to differences in the extent of initial neural damage and the underlying biological mechanisms of nerve repair.

A key finding of the present study is that both baseline clinical severity and the extent of EMG denervation were independently associated with recovery time. Patients presenting with more severe facial impairment and more pronounced electrophysiological abnormalities experienced a significantly prolonged recovery course, emphasizing the critical role of the initial degree of neural injury in shaping recovery dynamics.

Observation of the natural course of BP represents the basis for the appropriate interpretation of the effects of any therapeutic intervention. A large, uncontrolled study including 1701 patients demonstrated that approximately 70% recovered spontaneously within 6 months without specific treatment, although recovery was assessed using criteria that differed from contemporary standardized grading systems [2].

In a randomized controlled study, Mats Engström and collaborators reported that patients receiving prednisolone were more likely to achieve complete facial recovery compared with untreated controls, with HB grade I observed in approximately 78% and 64% of patients, respectively. These results contributed substantially to establishing early corticosteroid administration as the standard therapeutic approach for BP [11,22].

A recent study demonstrated that early application of manual acupuncture combined with electroacupuncture may shorten the time to complete recovery, with higher recovery rates after 12 weeks compared with electroacupuncture alone (93.4% vs. 80.3%). Although the authors used propensity score matching to improve comparability between groups, the observational design still carries the possibility of residual confounding. Nevertheless, Kaplan–Meier and Cox proportional hazards analyses suggested that earlier intervention was associated with faster recovery (HR 1.505; 95% CI 1.028–2.404) and a greater proportion of complete recovery during the early follow-up period [23].

Our findings are generally consistent with these observations. In our cohort of patients receiving acupuncture, most patients achieved complete recovery, with the majority of recoveries occurring during the early phase of follow-up. The Kaplan–Meier curves demonstrated a steep initial recovery phase followed by a gradual plateau. Beyond describing recovery patterns, Cox proportional hazards modeling identified clinical and electrophysiological factors associated with delayed recovery, providing additional insight into risk stratification in Bell’s palsy. Prolonged recovery may have important clinical implications, as persistent facial dysfunction can negatively affect daily functioning and quality of life. Overall, these findings are consistent with the concept that recovery represents a gradual regenerative process rather than a simple dichotomous clinical outcome.

Baseline HB grade emerged as one of the strongest predictors of recovery in our cohort, consistent with previous studies emphasizing the prognostic importance of initial clinical severity in Bell’s palsy. Patients with milder facial weakness usually have more preserved nerve function, which helps them recover faster and more fully. On the other hand, higher HB grades likely mean there is more nerve damage, such as severe demyelination and axonal injury, which slows down nerve repair and delays recovery.

Our findings also support that the starting severity of paralysis is a key factor in predicting recovery from Bell’s palsy. Recent studies show that patients with milder symptoms at the start are much more likely to recover well than those with more severe symptoms (82.9% vs. 68.2%, *p* < 0.001). This suggests that there are important differences in the extent of nerve damage and in how well it can heal [24].

What is the reason why EMG predicts the time to recovery independently, and CMAP does not? This difference may be explained by the fact that EMG denervation more directly reflects axonal injury and ongoing muscle denervation, whereas CMAP amplitude may additionally be influenced by transient conduction abnormalities and baseline clinical severity. Previous investigations assessing early electrophysiological predictors of recovery suggested that electroneurography and blink reflex testing may provide greater short-term prognostic utility than needle EMG [25].

However, these studies mainly evaluated short-term outcomes and performed electrophysiological testing very early after symptom onset, when EMG signs of denervation may not yet be fully apparent. In our cohort, a higher degree of EMG denervation remained associated with slower recovery during longer follow-up, possibly because it more directly reflects axonal damage and subsequent nerve regeneration.

More pronounced EMG denervation was independently associated with slower recovery, further emphasizing the importance of axonal injury in determining disease progression and regenerative capacity. In contrast, although lower baseline CMAP values were associated with poorer outcomes in univariable analyses, this association did not remain significant after adjustment for clinical severity and EMG findings.

These findings suggest that CMAP primarily reflects the overall extent of facial nerve injury rather than functioning as a fully independent prognostic marker. Its prognostic relevance appears to depend substantially on the broader clinical and electrophysiological context, particularly baseline HB grade and the degree of denervation. From a practical perspective, this indicates that isolated interpretation of CMAP values may be insufficient without integration with other clinical and neurophysiological parameters.

Our results nevertheless support the important role of electrophysiological assessment in prognostic evaluation of Bell’s palsy. Previous studies demonstrated that reduced CMAP amplitudes and pathological electrophysiological findings are associated with unfavorable outcomes [26]. The application of time-to-event analysis in our study additionally demonstrated its relationship with recovery dynamics over time. This approach provides further insight into disease progression and may contribute to improved clinical monitoring and risk stratification of patients with delayed recovery.

One of the more important findings of this study was the persistence of electrophysiological abnormalities despite complete clinical recovery. Although all analyzed patients achieved HB grade I, residual denervation signs on EMG remained present in the majority of patients. In addition, CMAP values frequently remained below expected physiological levels, suggesting that restoration of facial nerve function may occur without complete neurophysiological normalization.

These findings suggest that recovery may involve compensatory mechanisms beyond complete structural regeneration of the facial nerve. Partial reinnervation, collateral sprouting, and motor unit remodeling may contribute to the restoration of facial symmetry and voluntary movement despite persistent subclinical electrophysiological abnormalities. This discrepancy between clinical and neurophysiological recovery appeared particularly pronounced in patients with more severe initial paralysis, further supporting the association between the extent of axonal injury and long-term regenerative changes [27,28].

Acupuncture continues to be investigated as a potential treatment for Bell’s palsy. Previous systematic reviews have suggested potential benefits of acupuncture for functional recovery. However, the overall strength of the available evidence remains limited due to methodological heterogeneity, relatively small study populations, inadequate blinding, and substantial risk of bias in many published studies [13]. Nevertheless, several clinical investigations and meta-analyses have reported associations between acupuncture treatment and improved functional outcomes, although interpretation of these findings remains limited by methodological constraints [29,30].

Neuroimaging studies have reported associations between acupuncture and changes in activity within brain regions involved in facial motor control and sensory processing, although the clinical significance of these findings remains uncertain [29]. Several hypotheses have been proposed, including potential effects on inflammatory signaling, regional blood flow, oxidative stress, neurotrophic factors, and cortical reorganization; however, these mechanisms remain incompletely understood [31].

Although the observational nature of our study does not permit conclusions about therapeutic efficacy, the recovery outcomes observed in this cohort provides additional insight into recovery dynamics and prognostic factors among patients treated with acupuncture. Further well-designed randomized controlled trials incorporating standardized electrophysiological and longitudinal outcome measures are required to more clearly define the therapeutic role of acupuncture in Bell’s palsy.

Because all patients in this cohort received acupuncture, the study design does not allow clear differentiation between the effects of therapy and the disease’s natural course. Therefore, our findings should primarily be interpreted as describing the dynamics of recovery and identifying prognostic factors associated with disease outcome.

This study has several limitations that should be considered when interpreting the findings. The study was conducted as a single-center observational cohort without a control group, which limits the ability to draw definitive conclusions about the specific therapeutic effect of acupuncture. Consequently, it is not possible to distinguish treatment-related effects from the natural course of Bell’s palsy or to determine the specific contribution of acupuncture to the observed recovery outcomes. In addition, the exclusion of patients previously treated with corticosteroids or antiviral agents resulted in a selected cohort that may not fully represent the broader population of patients with Bell’s palsy encountered in routine clinical practice. Therefore, the findings of the present study should be interpreted primarily as describing recovery dynamics and prognostic factors within an acupuncture-treated cohort. Because treatment allocation was not randomized, the possibility of selection bias and residual confounding cannot be fully excluded.

Recovery was defined as House–Brackmann grade I, which represents complete clinical restoration of facial function. However, this outcome measure may not fully capture subtle subjective symptoms or persistent electrophysiological abnormalities and, therefore, reflects complete clinical rather than complete neurophysiological recovery.

Another important limitation relates to the electrophysiological assessment itself. Since EMG and CMAP recordings were obtained as part of routine longitudinal follow-up, variability in examination timing may have influenced the neurophysiological findings and their prognostic interpretation. In addition, all patients were treated in a specialized clinical setting, which may reduce the generalizability of the results to other healthcare environments and patient populations.

Recovery was defined as the first assessment at which House–Brackmann grade I was documented. Therefore, the exact date of recovery may have occurred between two consecutive assessments, and some degree of interval censoring cannot be excluded.

Assessment of Schoenfeld residuals indicated a modest deviation from the proportional hazards assumption for baseline HB category, suggesting that the effect of initial facial nerve dysfunction on recovery may vary over time. However, graphical assessment of Schoenfeld residuals and Kaplan–Meier curves showed no substantial departures from proportionality or crossing of survival curves, and the direction of the association remained consistent throughout follow-up. Therefore, the Cox model was considered appropriate for identifying prognostic factors, although the estimated effect of baseline HB category should be interpreted with appropriate caution.

Despite these limitations, the study also has several important strengths, including the large number of patients analyzed, prospective longitudinal follow-up, repeated clinical and electrophysiological evaluations, and the use of survival analysis to assess recovery dynamics over time.

## 5. Conclusions

The results of our study indicate that recovery from Bell’s palsy is a dynamic, heterogeneous process in which initial clinical severity and the degree of EMG denervation significantly influence the time required for complete functional recovery. More severe initial clinical deficits and more pronounced denervation were independently associated with slower recovery, confirming the prognostic value of combined clinical and electrophysiological assessment in the early phase of the disease. Although lower CMAP values were associated with prolonged recovery in univariate analysis, this association did not remain significant after adjustment for other parameters.

Persistent electrophysiological abnormalities observed in a subset of patients despite complete clinical recovery suggest that complete recovery may precede full neurophysiological regeneration of the facial nerve. These findings raise questions about the temporal course and extent of complete electrophysiological recovery, which may be a topic for future research.

The obtained results may contribute to more precise prognostic assessment, individualized follow-up, and improved treatment strategies for patients with Bell’s palsy, particularly by enabling earlier identification of patients with unfavorable prognostic factors and the need for more intensive monitoring and rehabilitation. Given the absence of a control group, these findings should be interpreted as describing recovery patterns and prognostic factors within an acupuncture-treated cohort rather than providing evidence of acupuncture efficacy.

## Figures and Tables

**Table 1:** Baseline clinical and electrophysiological characteristics of recovered and non-recovered patients.

Variable		Recovered (*n* = 843)	Non-Recovered (*n* = 207)	*p*-Value
Age (years)		41 [30–52]	56 [48–63]	<0.001 ^a^
Male sex, *n* (%)		483 (57.3%)	105 (50.7%)	0.103
HB *n* (%)	II	57 (6.8%)	0 (0%)	<0.001 ^b^
	III	281 (33.3%)	6 (2.9%)	
	IV	362 (42.9%)	18 (8.7%)	
	V	115 (13.6%)	149 (72.0%)	
	VI	28 (3.3%)	34 (16.4%)	
SB score		39 [26–57]	20 [14–32]	<0.001 ^a^
CMAP (%)		34 [23–50]	15 [8–22.5]	<0.001 ^a^
EMG *n* (%)	0	17 (2.0%)	0 (0%)	<0.001 ^b^
	1	53 (6.3%)	0 (0%)	
	2	310 (36.8%)	9 (4.3%)	
	3	245 (29.1%)	24 (11.6%)	
	4	218 (25.9%)	174 (84.1%)	

Legend: HB—House–Brackmann; SB—Sunnybrook; CMAP—Compound Muscle Action Potential; EMG—Electromyography. Data are presented as median (IQR) or *n* (%). Statistic test: ^a^—Mann–Whitney U test; ^b^—Chi-square test.

**Table 2:** Predictors of time to complete recovery: univariable and multivariable Cox regression.

Variable	Univariable HR (95% CI)	*p*	Multivariable HR (95% CI)	*p*
HB V–VI vs. HB II–IV	0.09 (0.07–0.12)	*p* < 0.001	0.12 (0.09–0.16)	*p* < 0.001
Male sex	1.55 (1.30–1.85)	*p* < 0.001	0.88 (0.73–1.07)	*p* = 0.192
Age (per year)	0.97 (0.96–0.97)	*p* < 0.001	0.98 (0.97–0.99)	*p* < 0.001
Time to treatment (per day)	1.02 (0.97–1.07)	*p* = 0.429	1.01 (0.96–1.06)	*p* = 0.679
CMAP ratio (per 1%)	1.05 (1.05–1.06)	*p* < 0.001	1.002 0.996–1.008	*P* < 0.576
EMG denervation (per grade)	0.41 (0.38–0.44)	*p* < 0.001	0.65 (0.58–0.74)	*p* < 0.001

Legend: HR—hazard ratio; CI—confidence interval; CMAP—Compound Muscle Action Potential; EMG—Electromyography; HB—House–Brackmann. Hazard ratios were estimated using Cox proportional hazards regression analysis.

## Data Availability

The data analyzed in the current study are not publicly available due to privacy and ethical considerations but may be obtained from the corresponding author upon reasonable request.

## Supplementary Materials

The following supporting information can be downloaded at https://www.mdpi.com/article/10.3390/medicina62071248/s1, Table S1. Description of the acupuncture intervention according to the STRICTA 2010 recommendations; Table S2. Detailed needling protocol used in the present study; File S1: STROBE Statement.

## Abbreviations

The following abbreviations are used in this manuscript:

BP	Bell’s palsy
HB	House–Brackmann
SB	Sunnybrook
EMG	Electromyography
CMAP	Compound muscle action potential
