# Supplementary material for: Time to Complete Clinical Recovery and Its Predictors in Bell’s Palsy Patients Receiving Acupuncture: A Prospective Cohort Study
Source: Medicina (Kaunas). 2026 Jun 29;62(7):1248. doi: 10.3390/medicina62071248 (PMC13413483; doi:10.3390/medicina62071248)
Supplement: Supplementary file 1 [file medicina-62-01248-s001.zip › Supplementary File S1-STROBE_Statement.pdf]

STROBE Statement

Manuscript: "Time to Complete Recovery and Its Predictors in Bell's Palsy Patients Receiving Acupuncture: A Prospective Cohort Study"

Checklist type: Cohort study. Prepared for submission with the revised manuscript. Locations refer to the current clean manuscript section/table/figure names and should be checked against final journal page numbering after typesetting.

Section/topic	Item no.	Recommendation	Status	Location / comment
**Title and abstract**	**1**	Indicate the study design with a commonly used term in the title or the abstract. Provide an informative and balanced summary of what was done and what was found.	**Reported**	Title; Abstract. The title identifies the work as a prospective cohort study and the abstract states that the study was prospective, observational, and uncontrolled, with a balanced summary of methods, results, and conclusions.
**Introduction: Background/rationale**	**2**	Explain the scientific background and rationale for the investigation being reported.	**Reported**	The Introduction describes the clinical burden and natural course of Bell's palsy, current evidence supporting corticosteroid therapy, and the ongoing uncertainty regarding the role of acupuncture. It further highlights methodological limitations of previous acupuncture studies and the limited availability of prospective data on recovery dynamics and electrophysiological changes, providing the rationale for the present investigation.
**Introduction: Objectives**	**3**	State specific objectives, including any prespecified hypotheses.	**Reported**	End of Introduction. Objectives are to evaluate time to recovery and identify baseline clinical/electrophysiological predictors, with an exploratory assessment of electrophysiological findings at clinical recovery.
**Methods: Study design**	**4**	Present key elements of study design early in the paper.	**Reported**	Materials and Methods, opening paragraph. The study is described as a prospective, observational, uncontrolled cohort study.
**Methods: Setting**	**5**	Describe the setting, locations, and relevant dates, including periods of recruitment, exposure, follow-up, and data collection.	**Reported**	Materials and Methods. Conducted at the Clinic of Neurology, University Clinical Center of Vojvodina, Acupuncture Outpatient Clinic, from January 2017 to August 2025; follow-up continued until recovery or up to 180 days.
**Methods: Participants**	**6a**	Give the eligibility criteria, and the sources and methods of selection of participants. Describe methods of follow-up.	**Reported**	Materials and Methods; Inclusion Criteria; Exclusion Criteria; Treatment Protocol and Follow-Up. Eligibility criteria, referral/self-referral pathways, consecutive eligible patients, and repeated clinical/electrophysiological follow-up are described.
**Methods: Participants**	**6b**	For matched studies, give matching criteria and number of exposed and unexposed.	**Not applicable**	Not applicable. This was not a matched cohort study and included no exposed/unexposed comparison groups.
**Methods: Variables**	**7**	Clearly define all outcomes, exposures, predictors, potential confounders, and effect modifiers. Give diagnostic criteria, if applicable.	**Reported**	Clinical Assessment; Electrophysiological Assessment; Statistical Analysis. The primary endpoint was time to complete clinical recovery (HB grade I). Predictors included age, sex, time from symptom onset to treatment initiation, baseline HB grade, CMAP ratio, and EMG denervation grade.
**Methods: Data sources/measurement**	**8**	For each variable of interest, give sources of data and details of methods of assessment. Describe comparability of assessment methods if there is more than one group.	**Reported**	Clinical Assessment; Electrophysiological Assessment; Acupuncture Intervention Protocol. HB and Sunnybrook scales, CMAP ratio, needle EMG grading, timing of baseline and follow-up assessments, and standardized electrophysiological procedures are described.
**Methods: Bias**	**9**	Describe any efforts to address potential sources of bias.	**Reported**	Materials and Methods; Clinical Assessment; Electrophysiological Assessment; Follow-Up; Limitations. Consecutive eligible patients were considered, predefined eligibility criteria were used, standardized clinical/electrophysiological protocols were applied, and lack of blinding, selection bias, residual confounding, and absence of a control group are acknowledged.
**Methods: Study size**	**10**	Explain how the study size was arrived at.	**Reported**	Materials and Methods. No a priori sample size calculation was performed; the final sample size was determined by consecutive eligible patients meeting predefined criteria during the study period.
**Methods: Quantitative variables**	**11**	Explain how quantitative variables were handled in the analyses. If applicable, describe which groupings were chosen and why.	**Reported**	Clinical Assessment; Statistical Analysis. Continuous variables are summarized according to distribution; baseline HB was dichotomized into HB II–IV and HB V–VI for prognostic and survival analyses; CMAP was analyzed as a continuous percentage ratio; EMG denervation was analyzed as an ordinal grade.
**Methods: Statistical methods**	**12a**	Describe all statistical methods, including those used to control for confounding.	**Reported**	Statistical Analysis. Descriptive statistics, Shapiro-Wilk testing, Mann-Whitney U test, chi-square test, Kaplan-Meier analysis, log-rank test, univariable and multivariable Cox proportional hazards regression are described and Schoenfeld residuals.
**Methods: Statistical methods**	**12b**	Describe any methods used to examine subgroups and interactions.	**Reported**	Results; Statistical Analysis. Kaplan-Meier recovery curves were stratified by baseline HB severity groups (HB II–IV vs HB V–VI). Electrophysiological findings at recovery were compared according to baseline HB severity.
**Methods: Statistical methods**	**12c**	Explain how missing data were addressed.	**Reported**	Treatment Protocol and Follow-Up. Patients with incomplete follow-up data were excluded; participants without recovery during 180 days were censored at their last available clinical assessment.

Section/topic	Item no.	Recommendation	Status	Location / comment
**Methods: Statistical methods**	**12d**	If applicable, explain how loss to follow-up was addressed.	**Reported**	Treatment Protocol and Follow-Up; Statistical Analysis. Non-recovered participants were censored at the last available clinical assessment; incomplete follow-up data were excluded.
**Methods: Statistical methods**	**12e**	Describe any sensitivity analyses.	**Not reported / not performed**	No formal sensitivity analyses are reported. Proportional hazards assumptions were evaluated using Schoenfeld residuals and graphical inspection, and related limitations are discussed.
**Results: Participants**	**13a**	Report numbers of individuals at each stage of the study.	**Reported**	Results. A total of 1050 patients were included; 843 achieved complete clinical recovery and 207 did not. Non-recovered patients' final HB status is reported. Figure 1 illustrates study design and follow-up workflow.
**Results: Participants**	**13b**	Give reasons for non-participation at each stage.	**Partly reported**	Eligibility and exclusion criteria are described, and patients with incomplete baseline or follow-up data were excluded. A detailed numeric flow of excluded patients by reason is not fully reported.
**Results: Participants**	**13c**	Consider use of a flow diagram.	**Reported**	Figure 1. Study design and follow-up workflow is included.
**Results: Descriptive data**	**14a**	Give characteristics of study participants and information on exposures and potential confounders.	**Reported**	Results; Table 3. Baseline clinical and electrophysiological characteristics are provided for recovered and non-recovered patients, including age, sex, HB grade, SB score, CMAP, and EMG denervation.
**Results: Descriptive data**	**14b**	Indicate number of participants with missing data for each variable of interest.	**Partly reported**	Materials and Methods state that incomplete baseline clinical/electrophysiological data and incomplete follow-up data were excluded. Variable-specific missing-data counts are not presented because the analytical cohort included complete baseline data.
**Results: Descriptive data**	**14c**	Summarize follow-up time.	**Reported**	Abstract; Results. Median time to complete recovery was 40 days (IQR 30–60); follow-up was continued until complete recovery or up to 180 days.
**Results: Outcome data**	**15**	Report numbers of outcome events or summary measures over time.	**Reported**	Results. Complete clinical recovery occurred in 843/1050 patients (80.3%); 207/1050 (19.7%) did not recover by follow-up/censoring. Time-to-event recovery results are shown with Kaplan-Meier analysis.
**Results: Main results**	**16a**	Give unadjusted estimates and, if applicable, confounder-adjusted estimates and their precision. Make clear which confounders were adjusted for and why.	**Reported**	Results; Cox regression table. Univariable and multivariable HRs with 95% CIs and p values are reported. The multivariable model included variables with $p < 0.10$ in univariable analysis and clinically relevant variables; SB was not included to avoid collinearity with HB.
**Results: Main results**	**16b**	Report category boundaries when continuous variables were categorized.	**Reported**	Clinical Assessment; Results. Baseline HB grade was dichotomized as HB II–IV versus HB V–VI.
**Results: Main results**	**16c**	If relevant, consider translating estimates of relative risk into absolute risk for a meaningful time period.	**Not applicable / limited**	The primary analysis used time-to-event methods and HRs. Overall recovery proportion and median recovery time are reported; no absolute risk translation for a fixed time point is provided.
**Results: Other analyses**	**17**	Report other analyses done, such as subgroup analyses and sensitivity analyses.	**Reported**	Results section 3.1. Electrophysiological findings at clinical recovery were compared according to baseline HB severity; Kaplan-Meier curves were stratified by HB severity.
**Discussion: Key results**	**18**	Summarize key results with reference to study objectives.	**Reported**	Discussion, opening paragraphs. The key findings are summarized in relation to the study objectives, including the overall recovery rate, median time to complete recovery, and the identification of baseline HB grade, age, and EMG denervation as independent predictors of recovery. The persistence of electrophysiological abnormalities despite complete clinical recovery is also discussed.
**Discussion: Limitations**	**19**	Discuss limitations, taking into account sources of potential bias or imprecision. Discuss direction and magnitude of any potential bias.	**Reported**	Discussion, Limitations. The manuscript discusses single-center observational design, absence of a control group, no randomization, selected cohort without prior steroid/antiviral therapy, residual confounding, lack of blinding, variable timing of electrophysiological assessments, interval censoring, limited generalizability, and modest PH assumption deviation for HB.
**Discussion: Interpretation**	**20**	Give a cautious overall interpretation of results considering objectives, limitations, multiplicity of analyses, results from similar studies, and other relevant evidence.	**Reported**	Discussion; Conclusions. The interpretation is cautious and emphasizes that findings describe recovery dynamics and prognostic factors within an acupuncture-treated cohort rather than proving acupuncture efficacy.
**Discussion: Generalisability**	**21**	Discuss the generalisability/external validity of the study results.	**Reported**	Discussion, Limitations. The manuscript notes that treatment in a specialized acupuncture outpatient clinic and the selected cohort may limit generalizability to broader Bell's palsy populations and other healthcare settings.
**Other information: Funding**	**22**	Give the source of funding and the role of funders for the present study and, if applicable, for the original study on which the article is based.	**Reported**	Funding statement. The manuscript states that the research received no external funding.

Note: This checklist was completed from the clean revised manuscript. It does not replace final editorial page/line verification after the manuscript is formatted by the journal.

Abbreviations: BP - Bell's palsy; **HB** - House–Brackmann; **SB** - Sunnybrook Facial Grading System; **CMAP** - Compound Muscle Action Potential; **EMG** - Electromyography; **HR** - Hazard Ratio; **CI** - Confidence Interval; **IQR** -Interquartile Range; **PH** - Proportional Hazards.
