# Supplementary material for: Time to Complete Clinical Recovery and Its Predictors in Bell’s Palsy Patients Receiving Acupuncture: A Prospective Cohort Study
Source: Medicina (Kaunas). 2026 Jun 29;62(7):1248. doi: 10.3390/medicina62071248 (PMC13413483; doi:10.3390/medicina62071248)
Supplement: Supplementary file 1 [file medicina-62-01248-s001.zip › Supplementary_Table S2_Acupuncture_Protocol.pdf]

Table S2. Detailed needling protocol used in the present study

Acupuncture point	Side	Depth (mm)	Angle of insertion	Needle manipulation	Retention time
Fengchi (GB20)	Bilateral	10–20	30–45°	Brief rotation	20 min
Hegu (LI4)	Bilateral	10–20	60–90°	Lifting–thrusting	20 min
Tinggong (SI19)	Affected side	10–15	45–90°	Rotation	20 min
Zanzhu (BL2)	Affected side	5–10	10–20°	Rotation	20 min
Yintang (EX-HN3)	Midline	5–10	10–20°	Rotation	20 min
Sibai (ST2)	Affected side	5–10	45–90°	Rotation	20 min
Dicang (ST4)	Affected side	15–25	10–20°	Rotation	20 min
Jiache (ST6)	Affected side	10–25	45–90°	Rotation	20 min
Renzhong (GV26)	Midline	5–10	10–20°	Rotation	20 min
Heliao (LI19)	Affected side	5–10	10–20°	Rotation	20 min
Taichong (LR3)	Affected side	10–20	30–60°	Rotation	20 min
Neiting (ST44)	Affected side	5–15	30–45°	Rotation	20 min
