# Supplementary material for: Time to Complete Clinical Recovery and Its Predictors in Bell’s Palsy Patients Receiving Acupuncture: A Prospective Cohort Study
Source: Medicina (Kaunas). 2026 Jun 29;62(7):1248. doi: 10.3390/medicina62071248 (PMC13413483; doi:10.3390/medicina62071248)
Supplement: Supplementary file 1 [file medicina-62-01248-s001.zip › Supplementary_Table_S1_STRICTA.pdf]

Table S1. Description of the acupuncture intervention according to the STRICTA 2010 recommendations

STRICTA Item	Description in the Present Study
1a. Style of acupuncture	Medical acupuncture.
1b. Reasoning for treatment provided	Treatment was based on a standardized protocol routinely used in the Acupuncture Outpatient Clinic for patients with acute Bell's palsy and informed by contemporary literature and clinical experience.
1c. Extent to which treatment was varied	A fixed treatment protocol was applied to all participants.
2a. Number of needle insertions per session	Twelve acupuncture points were used during each treatment session.
2b. Names of points used	Renzhong (GV26), Heliao (LI19), Dicang (ST4), Jiache (ST6), Sibai (ST2), Tinggong (SI19), Zanzhu (BL2), Yintang (EX-HN3), Fengchi (GB20), Hegu (LI4), Neiting (ST44), and Taichong (LR3).
2c. Depth of insertion	Needle insertion depth varied according to anatomical location and standard clinical practice.
2d. Responses sought	Needle manipulation was performed to elicit deqi sensation, characterized by heaviness, fullness, numbness, tingling, or a mild radiating sensation without significant pain.
2e. Needle stimulation	Manual acupuncture only. Electroacupuncture was not used.
2f. Needle retention time	Needles were retained for approximately 20 minutes during each treatment session.
2g. Needle type	Sterile disposable stainless-steel filiform needles (0.25 mm diameter; lengths 13–40 mm).
3a. Number of treatment sessions	Ten sessions per treatment cycle.
3b. Frequency and duration of treatment sessions	Three sessions per week. Treatment cycles could be repeated until complete recovery or for a maximum of 180 days.
4a. Other components of treatment	No additional acupuncture-related interventions were administered.
4b. Setting and context of treatment	Treatments were performed in a specialized outpatient acupuncture clinic according to a predefined protocol.
5. Practitioner background	Treatments were administered by physicians trained in medical acupuncture and experienced in the management of neurological disorders.
